# Automated Assessment of the Quality of Depression Websites

**DOI:** 10.2196/jmir.7.5.e59

**Published:** 2005-12-30

**Authors:** Kathleen M Griffiths, Thanh Tin Tang, David Hawking, Helen Christensen

**Affiliations:** ^4^Centre for Mental Health ResearchThe Australian National UniversityCanberraAustralia; ^3^ICT Centre, CSIROGPO Box 664CanberraAustralia; ^2^Computer Science DepartmentThe Australian National UniversityCanberraAustralia; ^1^Depression & Anxiety Consumer Research UnitCentre for Mental Health ResearchThe Australian National UniversityCanberraAustralia

**Keywords:** Quality indicators, depression, Internet, World Wide Web, validity, information retrieval

## Abstract

**Background:**

Since health information on the World Wide Web is of variable quality, methods are needed to assist consumers to identify health websites containing evidence-based information. Manual assessment tools may assist consumers to evaluate the quality of sites. However, these tools are poorly validated and often impractical. There is a need to develop better consumer tools, and in particular to explore the potential of automated procedures for evaluating the quality of health information on the web.

**Objective:**

This study (1) describes the development of an automated quality assessment procedure (AQA) designed to automatically rank depression websites according to their evidence-based quality; (2) evaluates the validity of the AQA relative to human rated evidence-based quality scores; and (3) compares the validity of Google PageRank and the AQA as indicators of evidence-based quality.

**Method:**

The AQA was developed using a quality feedback technique and a set of training websites previously rated manually according to their concordance with statements in the Oxford University Centre for Evidence-Based Mental Health’s guidelines for treating depression. The validation phase involved 30 websites compiled from the DMOZ, Yahoo! and LookSmart Depression Directories by randomly selecting six sites from each of the Google PageRank bands of 0, 1-2, 3-4, 5-6 and 7-8. Evidence-based ratings from two independent raters (based on concordance with the Oxford guidelines) were then compared with scores derived from the automated AQA and Google algorithms. There was no overlap in the websites used in the training and validation phases of the study.

**Results:**

The correlation between the AQA score and the evidence-based ratings was high and significant (r=0.85, *P*<.001). Addition of a quadratic component improved the fit, the combined linear and quadratic model explaining 82 percent of the variance. The correlation between Google PageRank and the evidence-based score was lower than that for the AQA. When sites with zero PageRanks were included the association was weak and non-significant (r=0.23, *P*=.22). When sites with zero PageRanks were excluded, the correlation was moderate (r=.61, *P*=.002).

**Conclusions:**

Depression websites of different evidence-based quality can be differentiated using an automated system. If replicable, generalizable to other health conditions and deployed in a consumer-friendly form, the automated procedure described here could represent an important advance for consumers of Internet medical information.

## Introduction

At least 50% of households in the United States, the United Kingdom and Australia are connected to the Internet [1-3]. In addition, many people have access to the Internet outside the home (eg, at work and in public libraries). A recent survey found that 79% of American Internet users have searched for health information online [4]. Moreover, there is evidence that online health information can improve health knowledge and health outcomes [5, 6].

To date, however, few health information websites have been subjected to rigorous assessment of their effectiveness in improving health outcomes. In the absence of such evidence, a minimum requirement for a high quality health information website should be that its content is evidence-based; that is, that its content and recommendations are consistent with evidence from a systematic review of the available medical literature. A non-evidence-based site would recommend treatments that are not supported by the evidence as effective, oppose the use of beneficial treatments of demonstrated benefit or fail to mention some effective treatments at all. For example, a depression webpage on the official site of the Church of Scientology claims that Dianetics is “the only proven effective technology of the human mind” [7] and fails to mention other medical, psychological and alternative treatments of demonstrated effectiveness. Elsewhere on the site, Zoloft and Prozac are described as “even more damaging than street drugs” [8].

Evidence-based health information is routinely disseminated to health professionals with the aim of assisting clinical decision making, improving healthcare and thereby improving health outcomes. Such evidence, when provided to consumers, has the potential to improve health outcomes by assisting consumers to select effective, rather than ineffective self-help techniques and by supporting shared decision making and consumer-provider collaborative care [9]. Unfortunately, reviews of the content of health websites have demonstrated that their quality, when assessed relative to evidence-based standards, is variable [10-13]. People seeking health information may therefore require assistance to filter out lower quality websites.

Various mechanisms have been proposed for assisting consumers to access high quality health websites [14]. These include the use of quality portals (such as OMNI in the United Kingdom and HealthInsite, Australia), pledges of webmasters to adhere to codes of conducts (such as the HON code [15]) and the use of consumer tools for assessing a site (for example DISCERN [16]). However, the criteria for inclusion in a quality portal typically do not include an evidence-based assessment, so their validity as guides to quality material is uncertain. Moreover, since such portals require time, effort and training to maintain, it may be difficult to update the database in a timely fashion. Similarly, codes of conduct and consumer tools employ accountability criteria (such as the identification of the author, their affiliations and qualifications) which are typically not validated against an evidence-based standard [11, 17, 18]. One exception is DISCERN, a tool designed to assist users without technical expertise to assess the quality of health information users. We have reported some preliminary evidence that DISCERN may be a valid indicator of the evidence-based quality of websites when used by consumers [19]. However, the tool may not be valid for all consumers. Moreover, DISCERN requires training and is lengthy, involving 15 items and requiring assessment of all the pages on the target website. In practice, individual consumers may lack the time and motivation to undergo the necessary training or to apply the tool to individual websites.

A potential solution to these problems is to develop assessment tools based on algorithms that automatically evaluate the quality of health information websites. To date there has been little work directed to this possibility. There is some evidence of a relationship between Google Page Rank and evidence-based quality from two recent studies [19, 20]. However, the association appears to be only of moderate strength, suggesting that a more valid automatic indicator of website quality may be required.

This paper describes the development of a computer algorithm, the Automatic Quality Assessment procedure (AQA), designed to automatically rank depression websites according to the evidence-based quality of their treatment information. In addition, it describes the results of an evaluation of the validity of the AQA as an indicator of human-rated evidence-based quality of treatment content. It also compares the validity of the AQA and Google PageRank as indicators of evidence-based quality.

The study focused on the evaluation of treatment information in depression websites since depression is a primary source of disability burden [21] and it has been reported to be a condition for which users commonly seek information on the Internet [4]. There is also a high degree of unmet need in the treatment of depression [22].

## Methods

This section comprises two parts. The first describes the AQA and its development. The second describes the methodology used for the validation of the AQA as an indicator of evidence-based treatment quality. The evidence-based rating scale [12] employed in developing and validating the AQA was based on clinical practice depression guidelines developed by the Centre for Evidence-Based Mental Health in Oxford from a systematic review of the evidence [23].

### The Automatic Quality Assessment Procedure (AQA)

In the following we present the procedure for calculating AQA scores and note its dependence upon two learned queries and three numerical parameters. We then describe the development phase during which the queries were learned and the parameter values chosen. The development phase employed websites/webpages not in the validation set but for which we had collected human-rated relevance or evidence-based quality measures from our previous studies [12, 19, 24].

The AQA assumes the availability of search engine software that incorporates a web crawler and has the ability to effectively score the relevance of documents to a query. The current study employed the Panoptic search engine for this purpose. However, we believe that other similar search engines could be substituted with similar results. A set of computer scripts were written to learn queries, to set values of tuning parameters and to collect and analyze output from the search engine. These scripts are not part of the Panoptic search engine.

### The Procedure

The AQA procedure comprised six steps as follows:

1. The target websites were downloaded using web crawler software;
2. These downloaded pages were aggregated with a large set of arbitrarily chosen general English language web pages and the resulting collection indexed using the search engine. This was necessary to avoid the extremely biased term frequency distribution of a depression-only collection;
3. A previously learned relevance query (see below) was processed over the collection created in Step 2 using the search engine to produce relevance scores for all documents. The relevance query consisted of many words and phrases, each with a numerical importance weighting. Documents with non-zero scores were not retrieved. For each site to be evaluated, the number of retrieved documents |R| were counted and the mean relevance score (*r*) computed.
4. A previously learned quality query (see below) was processed in the same fashion as in Step 3, yielding |Q| and *q*.
5. Site relevance and site quality scores were computed using Equations (1) and (2). These scores were normalized such that the highest S_r_ became 1.0 and the highest S_q_ was also 1.0.
6. An overall site score was computed using Equation (3). Gamma is a scaling parameter designed to make scores comparable with those from the human rating scale.

Equation 1: *S_r_*=*α* × *r* + (1-*α*)× |R|

Equation 2: *S_q_*= *α* × *q* + (1-*α*)× |Q|

Equation 3: *S* = *γ* × (*β* × *S_q_* + (1-*β*)×*S_r_*)

The following sections describe how relevance and quality queries were learned and the values of *α*, *β* and *γ* chosen.

### Learning Relevance and Quality Queries

Relevance and quality queries were learned using an extension and novel application of the relevance feedback technique from the field of information retrieval. In the relevance feedback approach, a complex query consisting of weighted terms (words and phrases), is automatically generated by comparing the term frequency distributions of sets of relevant and irrelevant documents. Good terms occur frequently in relevant text but seldom otherwise. The resulting query is used by a text retrieval system to derive relevance scores for documents. We extended this method to learn a 'quality' query from sets of high and low quality webpages.

*Relevance query:* During development of the relevance query, query terms were selected by computing Term Selection Values (TSVs) [25] for each candidate term, ranking them in descending order and taking all the terms above a cutoff. Numerical weights were applied to the selected terms using the Robertson-Sparck Jones formula [26].

Using 347 documents previously judged relevant to the topic of depression [24] and 9000 documents with very low probability of relevance to that topic, we generated a relevance query consisting of the words with the 20 highest TSVs and the two-word phrases with the 20 highest TSVs. The cutoff of 20 was arbitrary but consistent with past information retrieval practice.

*Quality query:* We generated a quality query in the same fashion, using 110 documents judged to be relevant to depression and of high quality as the “relevant” set and 3002 documents which were judged either irrelevant or relevant but not of high quality [24]. In this case the number of words (29) and phrases (20) in the query was the minimum number needed to ensure the inclusion of the names of all the evidence-based depression treatments listed in our previously published systematic review of the effectiveness of medical, psychological and alternative treatments for depression [27].

### Choosing Parameter Values

All the documents from 29 training sites for which we had human evidence-based (Oxford) ratings from previous studies [12, 19] were fetched using the Panoptic crawler and combined with 10000 documents from the Yahoo! Directory which were not in the depression category, as per Step 2 of the AQA procedure.

In following Steps 3 and 4 of the procedure during training, we computed |R|,*r*,|Q| and *q* based on scores obtained using the Okapi BM25 [28] relevance scoring mode of the Panoptic search engine. Okapi BM25 takes into account the frequency of occurrence of query terms in a document, the discriminating power of each query term, and the length of the document in calculating a relevance score.

The parameter adjusts the balance between the average document score and the coverage of a site. We then arbitrarily chose α = 0.75. The parameter β adjusts the balance between the relevance and quality scores for a site. We stepped through the range of values between 0.0 and 1.0 and chose the value which, when used in Equation 3, maximized the correlation between the computed site scores and the human-assigned quality scores. The best combination found, α = 0.75 and β = 0.70, yielded a correlation of 0.94 on the training data. It is possible that better values could be found with a more exhaustive optimization of parameters.

The parameter γ does not affect the correlation but scales the raw AQA scores to match the range of the human assigned scores. We chose γ = 17.27 which caused the highest AQA score to be the same as the highest human-rated score.

The values determined in training (α = 0.75, β = 0.70 and γ = 17.27) were used in the validity testing phase.

### Validity of the AQA versus PageRank

Here we describe the methodology used in a comparative validation study of the AQA and the Google PageRank procedures as an indicator of evidence-based treatment website quality. Each of two judges provided evidence-based ratings of 30 new depression websites. These ratings were compared with automated scores derived from the AQA and Google PageRank.

### Selection of Sites

The 30 depression information test websites were selected in the following manner.

First, we compiled a master list of all depression websites from the Open Directory (http://dmoz.org), Yahoo (http://www.yahoo.com) and LookSmart (http://www.looksmart.com) main and personal and treatment depression subdirectories as of September 2004. DMOZ, Yahoo and LookSmart are the three major human-compiled search engines on the World Wide Web. The human-compiled directories of many major crawler-based search engines such as Google are derived from Open Directory and the human-compiled content of the Lycos Directory is currently supplied by LookSmart.

After excluding websites that were no longer accessible, websites that were a subdirectory of an already included website, or “websites” that were actually links to an individual article, 208 websites remained.

Using the Google Toolbar, Google PageRank scores were recorded for each of these 208 websites. Sites were then pooled into 5 PageRank bands (0, 1-2, 3-4, 5-6 and 7-8) and, from each of the 5 PageRank bands, 6 websites were randomly selected (using a computer generated random number function) to form an initial set of 30 depression websites. Sites were stratified by PageRank prior to sampling to avoid generating a spuriously low correlation due to restricted range effects. A further 3 websites were excluded because the content was not free, there was no depression information on the primary site, or the site comprised only a single clinical tool for clinicians. These sites were each replaced from the equivalent PageRank band using the same computer generated random function.

Site content for each of the 30 websites was printed out in its entirety by systematically following all internal links. Any audio or video material content on a site was accessed online by the evidence-based raters and incorporated into the overall evidence-based score.

Content within a site was included for evaluation if it was free, written in the English language, comprised core informational material and focused on unipolar depression. Since the evidence-based rating scale employed in the current study was based on systematic guidelines for the treatment of major depressive disorder, pages in a site were excluded from evaluation if they focused on bipolar disorder, premenstrual syndrome, premenstrual dysphoric disorder or seasonal affective disorder. In addition, the following content on the target websites was excluded from the evaluation: news sections, videos of research conferences, book reviews, collections of PubMed abstracts, poetry, message board and chatroom content. This content was excluded because it was often unmanageably large (eg, poetry archives and chatrooms) and peripheral to the core educational material contained on the websites. General clinician assessment instructions and survey databases were excluded as they were not relevant to the study. Non-English content was excluded for practical reasons.

### Site Assessment

#### Site Characteristics

Test sites were coded independently by 2 raters according to their ownership structure (individual vs organization); whether or not they had an editorial board; whether or not the site was depression specific, was somewhat broader in scope, or comprised a clearinghouse; whether a health professional was involved; and whether the site promoted products or services (see Table 1). Where the two coders disagreed, the final categorization was assigned by a third rater (KG).

#### Evidence-Based Score

Each test site was rated independently by 2 raters using a 20-item rating scale previously developed by us [12] (see Textbox) from statements in the evidence-based, systematically developed clinical practice guidelines for the management of depression in primary care published by the Oxford University’s Centre for Evidence-Based Mental Health guidelines [23]. Only statements directly relevant to treatment were incorporated in the 20-item scale. The 30 test sites were rated in a different computer generated random order by the two raters. This rating scale has previously shown high interrater reliability [12, 19]. In the current study, interrater reliability was also very high (*r*=.93, *P*<.001) and there was no significant difference between scores for the two raters (mean difference= 0.17, 95% Confidence Interval (CI)= -0.96–0.62, *P*=.67).

**Evidence-Based Rating Scale for Human Raters**

The evidence-based rating scale [12] was developed from statements in the treatment section of *A systematic guide for the management of depression in primary care* published by the Centre for Evidence-based mental health, Oxford [23]

Antidepressant medication is an effective treatment for major depressive disorder.
Antidepressants are all equally effective.
The effectiveness of antidepressants is around 50 to 60%.
Full psychosocial recovery can take several months.
Drop out rate is same for different antidepressants.
The side effect profile varies for different antidepressants.
The choice of antidepressant should depend on individual patient factors (eg presence of co-morbid psychiatric or medical conditions, previous response to a particular drug, patient preference regarding the desirability of specific side-effects, concurrent drug therapy, suicidal risk)
Antidepressants are not addictive.
A trial of 6 weeks at full dose is needed before a drug can be considered to have failed and another tried.
A second-line drug should probably be from a different class of antidepressant.
Once improved continuation treatment at the same dose for at least 4-6 months should be considered.
Discontinuation syndrome may occur with abrupt cessation of any antidepressant so antidepressants should not be stopped suddenly. Where possible antidepressants should be withdrawn over a 4 week period, unless there are urgent medical reasons to stop the drug more rapidly. [To score 1, need to make general points that abrupt cessation can cause discontinuation syndrome and that antidepressants should not be stopped suddenly]
St John's Wort appears to be as effective as tricyclic antidepressants and causes fewer side effects, but little is known about any long term adverse effects.
Cognitive therapy can be an effective treatment for depression.
Cognitive behaviour therapy is at least as effective as drug treatment in mild-to-moderate depression.
Cognitive behaviour therapy may be valuable for people who respond to the concept of Cognitive behaviour therapy, prefer psychological to antidepressant treatment, have not responded to antidepressant therapy. [Score 1 if mention at least one of these]
Problem-solving may be effective for depression.
[Generic] counselling is probably no more effective than treatment as usual from the GP for depression.
Written information (usually based on a cognitive model of depression) can improve mild-to-moderate depression. [Score 1 if cognitive model]
Exercise can be effective - alone or as an adjunct to other treatments.

For each item, score 1 if the site information is consistent with the statement. Cumulate item scores across the scale to yield a total evidence-based score for the site.

#### Computing AQA Scores for the Test Sites

AQA scores were computed for the test websites by following Steps 1 to 6 of the AQA procedure using the relevance and quality queries and the values of α and β that were derived during training.

#### Google PageRank

The Google PageRank was recorded for each home page. Google PageRank is a measure employed by the Google Search Engine company to evaluate the reputation of a webpage. The PageRank is based on a computer algorithm that computes iteratively the number and importance of links to a webpage and in turn the number and importance of links to these linking pages [29]. As noted above, we identified the PageRank for each test site by downloading the Google Toolbar and recording the integer value (range 0 to 10) on the toolbar for the homepage of the site.

### Statistical Analysis

The sample size was considered sufficient to justify meaningful parametric analysis of the data. Intercorrelations between variables were computed using Pearson correlation tests. The validity of the automatic measure as an indicator of evidence-based quality was evaluated using hierarchical multiple regression. These analyses were performed using SPSS 12.0.1 [30]. Tests of the significance of differences between dependent correlations were computed using the SISA online calculator [31].

## Results

### Site characteristics

The characteristics of the 30 depression test sites used in the validation are summarized in Table 1. Two-thirds of the sites were depression-specific, a little over one-half were owned by an individual, and a health professional was involved in approximately half of the sites. One-fifth of the sites had an editorial board and over half of the sites promoted products or services or both.

**Table 1:** Characteristics of the websites employed in the test phase of the study

**Site characteristic**	**n (%) of sites (N=30)**
**Ownership structure**	
Individual	17 (56.7%)
Organization*	12 (40.0%)
Unknown	1 (3.3%)
**Editorial board**	
Yes	6 (20%)
No	24 (80%)
**Scope**	
Depression specific	20 (66.7%)
Broad scope	9 (30.0%)
Clearing house	1 (3.3%)
**Health professional involved**	
Yes	16 (53.3%)
No	14 (46.7%)
**Promotion of products/ services**	
Yes	19 (63.3%)
No	11 (36.7%)

^*^ Commercial, consumer or other organized group

### Quality scores

The mean (and standard deviation) of the evidence-based, Google PageRank and AQA scores were 5.92 (SD = 5.46; n = 30), 3.67 (SD = 2.59; n = 30) and 8.07 (SD = 5.22; n = 29) respectively. AQA scores were available for 29 test sites only as one website included a robots.txt exclusion, an indicator that the administrator of the site prohibited external crawlers from accessing the website.

**Figure 1:**
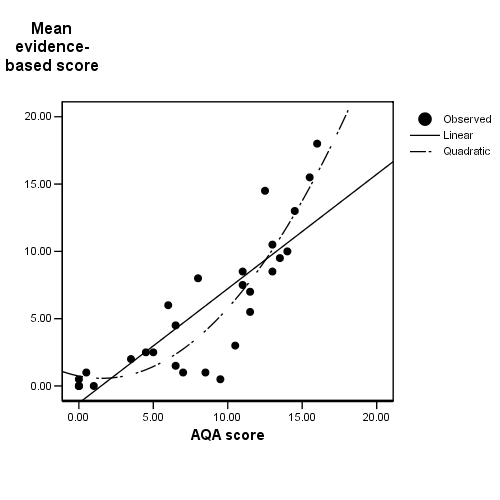
The relationship between the AQA and evidence-based scores

The relationship between the AQA score and the evidence-based ratings is shown in Figure 1. The linear correlation between these two measures was high and significant (r=0.85, *P* < .001).

**Table 2:** Summary of hierarchical regression analysis for predicting evidence-based quality from automatic quality

**Variable**	**B**	**SE(B)**	**β**	**P**
**Model 1**				
AQA	.85	.10	.85	*P*<.001
**Model 2**				
AQA	-.22	.30	-.22	*P* = .45
AQA^2^	.07	.02	1.11	*P* = .001

Note: Model 1: R^2^ = .71 ; Model 2: ΔR^2^ = .10 (*P* = .001)

Visual inspection of the scatterplot in Figure 1 indicated a possible quadratic component to the relationship. A hierarchical multiple regression analysis was therefore performed to determine if adding the square of the AQA score to the linear solution improved the prediction (see Table 2). A substantial 71.4% of the variance in the evidence-based quality score was explained by the automatic quality score alone. Addition of the quadratic component significantly improved the fit (∆R^2^ = 0.10, F∆ (1,26) = 14.3, *P* = .001), the combined linear and quadratic model explaining 82 percent of the variance.

**Figure 2:**
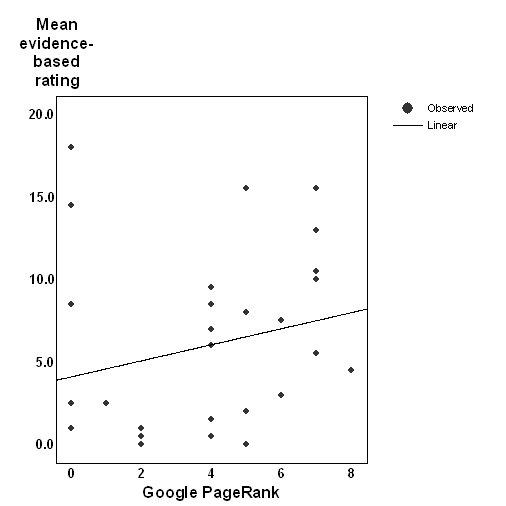
The relationship between the Google PageRank and evidence-based scores.

By contrast, the correlation between Google PageRank and the evidence-based score was small and non-significant (r = 0.23, *P* = .22, n = 30; see Figure 2). Excluding the missing case for which AQA could not be computed, this association between Google PageRank and the evidence-based score was significantly lower than the association between the AQA score and the evidence-based score (r(difference) (df = 26) = .64, t = 4.82, *P* = .0001).

Since it has been argued by some members of the search engine optimisation community that PageRanks of 0 may constitute a special subset of PageRank values (see Comment below), the above analyses were recomputed after excluding sites with PageRank of 0. The association between the PageRank and evidence-based scores for remaining sites was significant (r = 0.61, *P* = .002, n = 24), but remained significantly lower than the association between the AQA and evidence-based quality scores (r(difference) (df = 20) = 0.22, t = 2.61, *P* = .02).

## Discussion

### Principal Results

A recent article concluded that “quality benchmarking of health-related resources will always depend on a human assessor …” [32]. We have demonstrated that an algorithm based on relevance feedback (and involving no human judgment) is a valid indicator of evidence-based quality of the treatment content of depression sites. To our knowledge, this is the first published study of the validity of a custom designed automated tool for identifying the evidence-based quality of health information. If replicable and generalizable to other health conditions, the current findings may have major practical implications for e-health, consumer empowerment and self-managed healthcare.

Previous researchers have developed search systems designed to identify medicine-specific Web-based information [33]. However, these systems focus on identifying material that is relevant to the medical domain rather than selecting sites of high content quality.

One published study has described a prototype system for rank ordering Web-based health information by quality [34]. However, this paper used accountability criteria (eg, presence of authorship information, detection of an HONcode logo, detection that the page included information about the date it was last updated) rather than content accuracy as a benchmark of quality. There is little or no evidence that these accountability measures singly or together correlate with evidence-based content quality [10, 11, 17, 18]. Moreover, the researchers did not evaluate the content quality of the retrieved pages in order to validate their system of ranking against an evidence-based standard. Finally, in contrast to the procedure described in the current study, the system focused on individual pages rather than on the entire website on a topic. It may be that only by examining all the content of a site is it possible to gain a comprehensive picture of its quality.

The finding in this study that websites can be automatically evaluated for content quality is of considerable practical significance. Suitably adapted, refined and integrated into or used to post-process websites retrieved by a general search engine, this system could assist consumers to identify websites of higher quality. In the shorter term, the system can be used to compile lists of websites for use in a focused search engine for depression, such as that used on the BluePages Depression Information website (http://bluepages.anu.edu.au). In addition, the system may prove useful as a screening device for the use of web developers interested in maintaining quality health portals or links of high quality. Once filtered by the automatic quality evaluator, developers could evaluate the remaining sites manually to confirm accuracy of content, and to assess sites according to other dimensions of quality (eg, usability) and according to the needs of the organization and its users.

Consistent with previous studies [19, 20] we have demonstrated in the current study that Google PageRank may be an indicator of evidence-based quality. However, the fact that Google PageRank was unable to provide a meaningful quality assessment for sites with a zero score is a significant impediment to its practical use. Moreover, even with zero PageRanks excluded, the association between PageRank and content quality is less strong than the association between the AQA score and content quality. This suggests that relevance feedback (employed by our AQA) may be superior to link structure (employed by Google PageRank) as a method for identifying evidence-based quality for a specific health domain. It also provides evidence that a specialized tool such as the AQA is warranted. It might be argued that a combination of relevance feedback and link structure methods could improve the validity of the AQA further. However, the preliminary indications are that this is not the case. Supplementary analyses showed that when the Google PageRank was added into the regression model along with the AQA scores, it did not improve the fit of the model. This was the case whether zero page ranks were included (explained 71% of the variance compared to 82% in the original model) or excluded (explained 65% compared to 82%).

The current tool is relevant only for identifying the quality of depression websites. However, the relevance feedback method used in the AQA is likely to generalize to other areas of mental health and medicine. To apply the AQA in alternative health domains requires that the procedure is trained to learn new terms and parameter values specific to the new domain. The validity of the technique in these other domains is a matter for empirical investigation.

### Limitations

There are some limitations of the system described here. First, some minor changes to the AQA scaling procedure are necessary before the system is used in practice (particularly for evaluating a single website at a time). Applying a non-linear transformation to the raw AQA scores (rather than linear scaling) might result in a better prediction of the values of the evidence-based scores. However, given that the base (linear) correlation coefficient is already very high (0.85), the scope for improvement is limited. Secondly, the AQA scores could be compromised if publishers use “spamming” methods for optimizing their automatic quality scores. General search engines are faced with a similar problem when website developers attempt to artificially inflate the relevance rankings for their websites. This problem is not peculiar to automated methods for processing websites. It is likely that website developers also use strategies to maximize their scores on manually applied quality evaluation tools while leaving unchanged the substantive content. Public search engines incorporate algorithms for detecting attempts to distort rankings. It is likely that the AQA could be refined to do the same. The third limitation of the AQA system is that it is focused on treatment information (as indeed is DISCERN) and does not necessarily reflect the quality of other information on a site. In addition, the system may not adequately rate sites which present only one treatment type. The system will be most useful in identifying sites containing high quality, comprehensive treatment information. Another limitation relates to the gold standard employed for rating the evidence-based quality of the sites. It might be argued that to be considered valid, the rating system should be validated against health outcomes or another scale that has been thus validated. No such scales exist, and given the paucity of efficacy studies of websites, such validation exercises are not currently practical. Finally, one of the evidence-based raters had been involved in the initial selection of the sites which may have led to bias in the findings from the validity studies. However, the pattern of results and conclusions is identical if the findings are recomputed using the blinded rater’s data only. This is not surprising given the higher interrater reliability in this study for the evidence-based scale.

### Conclusion and Future Work

The time has come to acknowledge that consumers do and will continue to use the Internet as a source of health information. We need to provide them with convenient, effective tools that optimize the usefulness of this process. This study demonstrates that automated methods offer considerable promise in this respect. The task before us now is to refine these methods, and to test the usability, robustness and generalizability of the systems we develop. In the process we need to test alternative strategies for quality filtering, to identify if multiple methods when combined improve the validity of the automatic algorithm, and to evaluate whether the techniques generalize across health domains. We must also construct a user interface for the procedure, and conduct consumer user and satisfaction studies on the resulting system.

## Abbreviations

AQA: automated quality assessment procedure
TSVs: Term Selection Values
